# Coeliac disease and intestinal inflammation in juvenil idiopathic arthritis

**DOI:** 10.1186/1546-0096-9-S1-P168

**Published:** 2011-09-14

**Authors:** Esmeralda Nuñez, Rocio Galindo, Enrique Lopez, Gisela Diaz, Maria Isabel Vicioso, Carlos Sierra

**Affiliations:** 1 Carlos Haya Children Hospital, Malaga, Spain

## Background

Coeliac disease (CD) is an enteropathy that affects about 0,5-1% of the population. Even though it could be associated to diabetes, thyroiditis and Sjögren syndrome, there is not conclusive studies about its juvenile idiopathic arthritis (JIA) correlation. Testing for fecal calprotectin is an effective way to screen for inflammatory bowel disease. Protein-losing enteropathy screening using alpha-1 antitrypsin along with intestinal inflammation suggests severe intestinal mucosal damage.

## Aims

CD screening and evaluation of intestinal inflammation status in a cohort of JIA patients. Moreover to know the breast milk duration and if IgA deficiency exists.

## Methods

Sixty three children with JIA under 14 years old were evaluated. All they received systemic treatment. Inmunoglobulins, antitransglutaminase IgA (IgG if IgA deficiency) and anti-endomysium antibodies test were determined. Fecal calprotectin and fecal alpha-1 antitrypsin were investigated. Data from breast milk, digestive symptoms and anthropometric measurements were analized.

## Results

The average age was 7,8 years (48 female) and 3,9 years when they were diagnosed. JIA was defined as oligoarticular in 39 children (14 ANA+), polyarticular in 14, systemic in 6 and enthesitis related arthritis in 4 cases. Most frequent treatments were: methotrexate (36), methotrexate + biological therapy (21), biological therapy (3), prednisone (3), NSAIDs (3). Positive family history for autoimmune disease (bowel intestinal disease in 2 cases) was found in 41,3 %. 79% were fed with breast milk (6 months average). The most frequent digestive symtomps were: abdominal pain (16), constipation (7) and diarrhea (5). None was found IgA deficient. One patient was CD diagnosed before AIJ. Two asymptomatic children cases were positive for antitransglutaminase IgA and anti-endomysium antibodies, confirming CD by intestinal biopsy in one of them and the inmmunological test turned negative in the other case. Fecal calprotectin were over the limit (50 µg/g) in 28,5% (50-100 µg/g in 11 and over 100 µg/g in 7 children). Fecal alpha-1 antitrypsin was negative in all cases. Average anthropometric measurements: height Z score - 0,7 , weight Z score -0,37, BMI Z score -0,004. In 25,4% of patients weight Z score was <-1 and in 35% height Z score was <-1.

## Conclusions

Our study shows higher CD prevalence in JIA patients compared to healthy children, so even without digestive symptoms we consider screening test needed. Fecal calprotectin test over the limit indicates intestinal inflammations, so further investigations should be done especially for those patients without NSAIDs treatment in order to discard bowel intestinal disease.

